# Designing and piloting a generic research architecture and workflows to unlock German primary care data for secondary use

**DOI:** 10.1186/s12967-020-02547-x

**Published:** 2020-10-19

**Authors:** Thomas Bahls, Johannes Pung, Stephanie Heinemann, Johannes Hauswaldt, Iris Demmer, Arne Blumentritt, Henriette Rau, Johannes Drepper, Philipp Wieder, Roland Groh, Eva Hummers, Falk Schlegelmilch

**Affiliations:** 1 Institute for Community Medicine, Section Epidemiology of Health Care and Community Health, University Medicine Greifswald, Ellernholzstr. 1-2, Greifswald, 17475 Germany; 2 Department of Medical Informatics, University Medical Center Göttingen, Robert-Koch-Str. 40, Göttingen, 37075 Germany; 3 Department of General Practice, University Medical Center Göttingen, Humboldtallee 38, Göttingen, 37073 Germany; 4 Trusted Third Party of the University Medicine Greifswald, Ellernholzstr. 1-2, Greifswald, 17475 Germany; 5 TMF – Technology, Methods, and Infrastructure for Networked Medical Research (TMF e.V.), Charlottenstraße 42, Berlin, 10117 Germany; 6 Gesellschaft für wissenschaftliche Datenverarbeitung mbH, Am Faßberg 11, Göttingen, 37077 Germany

**Keywords:** Health information exchange, Primary health care, Electronic health records, Informed consent, Data management architecture, GDPR, Trusted third party, Medical record linkage, Secondary use

## Abstract

**Background:**

Medical data from family doctors are of great importance to health care researchers but seem to be locked in German practices and, thus, are underused in research. The RADAR project (Routine Anonymized Data for Advanced Health Services Research) aims at designing, implementing and piloting a generic research architecture, technical software solutions as well as procedures and workflows to unlock data from family doctor’s practices. A long-term medical data repository for research taking legal requirements into account is established. Thereby, RADAR helps closing the gap between the European countries and to contribute data from primary care in Germany.

**Methods:**

The RADAR project comprises three phases: (1) analysis phase, (2) design phase, and (3) pilot. First, interdisciplinary workshops were held to list prerequisites and requirements. Second, an architecture diagram with building blocks and functions, and an ordered list of process steps (workflow) for data capture and storage were designed. Third, technical components and workflows were piloted. The pilot was extended by a data integration workflow using patient-reported outcomes (paper-based questionnaires).

**Results:**

The analysis phase resulted in listing 17 essential prerequisites and guiding requirements for data management compliant with the General Data Protection Regulation (GDPR). Based on this list existing approaches to fulfil the RADAR tasks were evaluated—for example, re-using BDT interface for data exchange and Trusted Third Party-approach for consent management and record linkage. Consented data sets of 100 patients were successfully exported, separated into person-identifying and medical data, pseudonymised and saved. Record linkage and data integration workflows for patient-reported outcomes in the RADAR research database were successfully piloted for 63 responders.

**Conclusion:**

The RADAR project successfully developed a generic architecture together with a technical framework of tools, interfaces, and workflows for a complete infrastructure for practicable and secure processing of patient data from family doctors. All technical components and workflows can be reused for further research projects. Additionally, a Trusted Third Party-approach can be used as core element to implement data privacy protection in such heterogeneous family doctor’s settings. Optimisations identified comprise a fully-electronic consent recording using tablet computers, which is part of the project’s extension phase.

## Background

Medical data from family doctors (FD) are of great importance to health care researchers but seem to be locked in the FD’s practice (FP) and, thus, are underused in research. Unlocking routine data from FPs can provide an essential source of information for health services, medical research as well as health policy decisions. Further, making this data source available offers the possibility to link it with secondary care data or additional data obtained from other research sources—for example, patient-reported data. The RADAR project (Routine Anonymized Data for Advanced Health Services Research) aims at designing, implementing and piloting a generic research architecture, technical software solutions as well as procedures and workflows to unlock data from FD practices and establish a long-term medical data repository for research, whereby the legal framework is also taken into account.

Architectural building blocks and technical solutions are already available for clinical and clinical-epidemiological research, but were not considered and evaluated if and how they can be applied and reused for FPs’ data yet. There are only few representative and longitudinal data from primary care or scientific cross-regional studies in family medicine available in Germany compared to other European countries. For example, Denmark established a comprehensive national registry [1, 2], the United Kingdom uses an extensive primary care data base derived from routinely recorded electronic health records (EHR) since 1987 [3], and Sweden built a nationwide quality assurance system of all primary healthcare centres [4].

In Germany, this is mainly due to the fact that in FDs’ practices existing technical structures are insufficiently equipped to carry out medical research. FDs’ practice management systems (PMS) are not only numerous and heterogeneous with about 165 systems on the market (as of market analysis in Q1/2018) [5] but also hardly support the exchange of data. Although a standard for the exchange of medical data was established by the Kassenärztliche Bundesvereinigung (KBV; English: Association of Statutory Health Insurance Physicians) in 1994, it is not uniformly implemented in PMS. A few evaluations of family practice (FP) data available in Germany emphasise this problem: Most German PMS offer only an outdated interface for data transfer (German: Behandlungsdatentransfer-Schnittstelle, BDT) [6], which in most cases is locked by an additional license or only accessible by personnel of the vendor. Efforts to use this BDT interface as a data source for research were first made in 2001–2003 [7]. Within this study, FDs received an export instruction for each PMS and a set of blank floppy-disks by post. The exported BDT data were anonymised on-site in the FD’s practice and sent back by post—thus, leaving the FD’s practice. The findings of this generic study resulted in the use of BDT data export for the MedViP project [8]. In MedViP, the data transfer path of encrypted BDT-files was extended from (a) by post to the further possibilities of (b) email, (c) web upload and (d) delivery in person [9]. Based on the BDT data collected within the MedViP project, several health services research questions were answered and the results published [10–14]. Also, BDT is a basic building block of the BeoNet Registry Database, extended by further data exchange formats created in cooperation with the KBV and implemented in cooperation with a software vendor [15]. The BeoNet Registry-Database uses encrypted transmission of pseudonymous BDT data via a Virtual Private Network (VPN) connection.

The RADAR project aims to close the gap between the European countries and to contribute and pilot a generic research architecture featuring representative data from primary care in Germany. To achieve the set aims, the RADAR project (1) analyses technical and legal prerequisites and requirements, (2) designs and implements a generic technical research architecture as well as workflows by identifying building blocks to unlock FPs’ data and (3) tests the project results in a pilot using follow-up questionnaires and linking the questionnaire data to FPs’ data.

## Methods

The RADAR project’s work to accomplish the set aims has been divided into three phases: (1) an analysis phase, (2) a design phase, and (3) a pilot.

### Phase 1: analysing prerequisites and requirements

To identify prerequisites and requirements, multiple interdisciplinary workshops with FDs, experienced researchers, medical data managers as well as software architects and developers, data security experts and TMF staff (TMF—Technology, Methods, and Infrastructure for Networked Medical Research) were conducted. Project workshops were designed as one-day meetings with the same participant groups—the so-called RADAR project team—and were conducted using the formats face-to-face, telephone or web meeting. Additionally, the participating FD sites were visited and on-site practice staff included in discussions regarding the practical implementation.

The result of this project phase was a list of prerequisites and requirements.

### Phase 2: designing architecture and workflows

During phase 2, the workshop format described in phase 1 was continued but re-focussed.

First, existing architectural approaches and existing functions to meet the in phase 1 collected requirements were analysed by reviewing available literature [15–19]. Additionally, not-yet-published architectures of current projects that the partners participate in, such as the scientific infrastructure of the German Centre for Cardiovascular Research (DZHK), were considered.

Second, building blocks and functions needed to complete the RADAR tasks of

- Exporting data from PMS,
- Recording and checking a patient’s consent for RADAR project,
- Storing patient’s identifying data (IDAT) and respective RADAR consent, generating pseudonym, and storing the association between identifying data and pseudonym,
- Pseudonymising the EHR data subset for which a RADAR consent exists, and
- Transferring the pseudonymised data into the RADAR research database for later data use

were modelled consistently with the findings of the first step. Expertise among the RADAR project partners was considered when distributing RADAR’s tasks with regards to building blocks and functionalities required:

- Data privacy protection expertise of the partner TMF.
- Available solutions for data privacy protection using the Trusted Third Party (TTP) from the University Medicine Greifswald, including an interface specification [20], already used in several current projects.
- In-depth knowledge of data handling and processing, e.g. in the DZHK, at the Department of Medical Informatics, University Medical Center Göttingen.
- Research database knowledge with regards to data storage and accessibility at the GWDG (Gesellschaft für wissenschaftliche Datenverarbeitung mbH in Göttingen).
- Comprehensive insight in FP procedures from medical and practice management perspective at the Department of General Practice of the University Medical Center Göttingen.

Third, data flows through the architecture were discussed and modelled among all project partners. Processes modelled in this early project phase have been limited to data capture, transfer, and storage as well as search and retrieval within the research data. All intermediate steps of the processes were documented, together with the respective ‘source’ and ‘destination’ building block, and the function carried out. Thereby, a high-level interface definition was deducted.

The result of this project phase was an architecture diagram with building blocks and functions that mapped onto the organisational structure of the RADAR project, and an ordered list of process steps (workflow) for data capture and storage.

### Phase 3: implementing, testing and piloting

Based on the results of phase 1 and 2, the RADAR building blocks and functions were implemented, tested and piloted in phase 3. This process is divided into two consecutive parts: first, implementation and testing of technical RADAR components and, second, piloting of the RADAR architecture and workflows—extended by a paper-based questionnaire to demonstrate data linkage processes.

The implementation of the technical RADAR components (i.e., building blocks and functions) focussed on the processes for exporting, selecting and transferring data from FDs’ PMS as well as data linkage and integration to allow search and retrieval of research data. During the technical implementation, component-based software tests were conducted continuously. Afterwards, tests focussed on the connection between and interaction of software-based architectural building blocks. At the end of the implementation part, scenario tests (i.e., workflows) were done to check for functionality and feasibility barriers—first, within a test environment and, second, in a selected participating FD’s practice as real-world scenario.

After all tests were successfully completed, the RADAR architecture comprising technical components as well as workflows was piloted. The aim of this pilot was to successfully export, transfer and save FPs’ data sets of participating patients into the pseudonymised RADAR research database.

Afterwards, a paper-based follow-up questionnaire was developed for patients of participating FPs. The purpose of this questionnaire was to pilot the workflow of integrating data of paper-based patient-reported outcomes directly into the RADAR research database, as well as linking these data with existing patients in the RADAR research database.

## Results

### Phase 1: analysing prerequisites and requirements

As a result of the interdisciplinary workshops, the RADAR project team has identified a number of prerequisites and guiding requirements (short: requirements) for the desired project and the respective data management compliant with the General Data Protection Regulation (GDPR). These requirements are listed in the following Table 1. The list starts with FD-associated requirements (no. 1–6), followed by technical requirements for data export, transfer, and storage (no. 7-10). Furthermore, a few overall requirements (no. 11, 14–17) are results of the interdisciplinary workshops as well.

**Table 1:** List of prerequisites and requirements (referred to in the following text as “REQ”)

REQ No.	Prerequisite/Requirement
1	As FD practices focus on every-day primary health care of walk-in patients, additional (research) activities, time and workload for the practice staff must be reduced to a strict minimum. This means, daily or continuous operation of processes to support the RADAR project must be avoided wherever possible
2	Practice staff may not have the necessary technical knowledge and may need local support and direct assistance by IT-staff from the RADAR project
3	Data should be extracted from the patient’s health record as automatedly as possible
4	Data source is the FP’s PMS, i.e. so-called routine data. As a result, FD practices that work mostly paper-based are excluded from the RADAR project
5	No hardware or any additional software may be implemented into or reconfigured in the PMS. If the PMS is adjusted in any way, the FP may lose warranty or product support by the PMS or hardware supplier in case of problems with the PMS
6	Data extraction from the PMS should not be scheduled during peak office hours. Rather, data extraction, transformation and loading must be done during time periods without or with minimum patient traffic
7	The PMS’s export interface must be accessible for use
8	Compound data sets including person-identifying data [IDAT] and medical data [MDAT] must not leave the practice. Rather, data must be split into IDAT and MDAT within the practice before being transferred to the RADAR research domain
9	The RADAR data transfer solution should deal with as-is internet access availability of the practices
10	The RADAR data export should deal with local practice-internal network availability
11	Patient’s consent and release of obligation to maintain confidentiality is basis for legal data processing, and must be a design element for the operations model
12	The RADAR project team should provide a RADAR-specific consent, patient information and release of obligation to maintain confidentiality
13	The RADAR project team manages processes with ethics committees and provides a positive vote
14	The RADAR consent recording must be (a) convenient for practice staff as well as (b) patients but still (c) digitalised as early as possible in the process
15	RADAR project provides a data protection concept based on the guidelines of the TMF. This includes the integration of a TTP for the administration of identifying data and assigned pseudonyms
16	RADAR work packages and building blocks of the solution must (a) map to the number and expertise of project partners and still provide a setup that (b) complies with GDPR requirements
17	RADAR solution should allow to select and export data subsets from the research database in a web-based, easy-to-manage way for subsequent analysis

Summarising, the interdisciplinary project team concluded that unlocking primary care data for secondary use in a FP setting has significant differences from a hospital setting. Examples for such differences comprise documentation standards (paper, computerised, unstructured text vs. structured items), network availability (internet access, practice-internal network, non-networked PCs), heterogeneity of PMS used, and legal framework conditions [21]. Besides such rather formal aspects and compared with clinical settings, FP staff is less experienced and trained in informing patients about secondary use of medical data for research and procedures regarding the recording of patient’s informed consent. This leads to respective requirements for the RADAR project team (no. 12–13).

### Phase 2: designing architecture and workflows

Based on the above listed prerequisites and requirements the design process for RADAR architecture and workflows began with evaluating existing approaches to fulfil the RADAR tasks. Reviewed literature led to the conclusion that using the BDT data export interface for exporting EHR data was one of the cornerstones of the earlier BeoNet project in a similar FP setting [15]. The possibility to export EHR data successfully using the BDT interface was demonstrated. Although the BDT interface is outdated, it’s still the only available interface for data export in German PMS. Therefore, the RADAR architecture will re-use this concept and also base its data export on the BDT interface (REQ #7, #3, #2). However, subsequent processes in MedViP using floppy disks and postal service for data transfer will not be re-used.

To store medical data including those originating from follow-ups in a research database for scientific use has been described by the German National Cohort (GNC) [16]. RADAR and GNC share the approach to collect data sets which are not per se limited to a pre-set research question but rather to collect a broader data set to allow for later definitions of research hypothesis and their tests. Specifics of the GNC solution were discussed but are not re-used for RADAR as the settings of GNC and RADAR differ (study setting with appointments for participants without medical treatment (GNC) vs. FD practice with clear focus on medical treatment of patients (RADAR), REQ #1).

In contrast to the GNC setting, the DZHK scientific infrastructure supports a study setting in real-life clinical situations [22] with medical treatment of patients and seems a better fit to consider for modelling workflows. However, both GNC and DZHK share the same architectural building block of a Trusted Third Party (TTP) for GDPR-compliant data processing. In both settings a TTP [17] is used to manage IDAT while the research database only contains pseudonymised medical data (REQ #8, #11). This approach allows also for automated queries regarding real-time consent states and data usage policies, e.g. for subsequent data usage. Furthermore, the TTP supports automated record linkage procedures for existing data and for usage of additional data sources: IDAT of the new dataset are matched with all existing RADAR participants to link data of the same person originating from different data sources (e.g., a second FP). Such record linkage procedures consider spelling ambiguities, typos, or changes of names, thereby reducing the number of duplicates in research data. Functions needed for RADAR were already indicated in the methods chapter, phase 2, (a) to (e). Literature review and workshops led to the conclusion that these functions correspond with the following architectural building blocks and their operation in the RADAR project:

- PMS holding all patients’ EHR data of a respective FD practice in digital format including the BDT data export interface. This interface needs to be unlockable to allow export of all patients’ EHR data (accounted for by the Department of General Practice of the University Medical Center Göttingen, RADAR project partner A; REQ #7, #3, #2).
- RADAR software module that supports itemised digital data entry for the consent information and its status per patient (implemented by the Department of Medical Informatics of the University Medical Center Göttingen, RADAR project partner B; REQ #8, #14, #11).
- TTP with software modules and interfaces to i) communicate and store IDAT of patients, who participate in RADAR, ii) store and display the RADAR consent text, iii) store each individual patient’s consent status per consent module or item, iv) generate unique pseudonyms for each RADAR patient, and v) store the association between IDAT and pseudonyms (provided by the Trusted Third Party of the University Medicine Greifswald, RADAR project partner C; REQ #8, #11).
- RADAR software module that pseudonymises the EHR data subset for which a RADAR consent exists (implemented by the Department of Medical Informatics of the University Medical Center Göttingen, RADAR project partner B; REQ #8, #14).
- RADAR research database with data import and export interface as well as web-based user interface to submit search queries and trigger exports (implemented by the GWDG in Göttingen, RADAR project partner D; REQ #17).

**Fig. 1:**
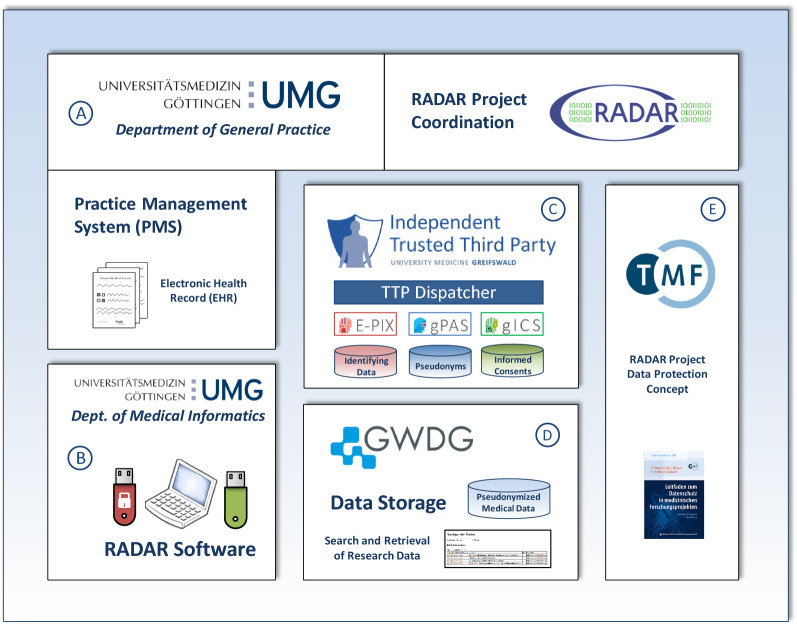
Project partner organisation and mapped architectural building blocks

During a workshop among the RADAR stakeholders, it was agreed to combine the software module (b) with (d). As the data set originating from (b) is one of the input data for operation in (d), a functional combination in one single RADAR software module seemed advisable. Project implementation and operation was organised according to this blueprint afterwards (REQ #16). The RADAR project lead is at the Department of General Practice of the University Medical Center Göttingen. The TMF (RADAR project partner E) supported the RADAR project with advice on data protection measures and options, and discussed and drafted the data protection concept (REQ #15). Figure 1 shows the organisation of the respective project partners in RADAR and the mapped architectural building blocks they were responsible for during project implementation (operational perspective).

**Fig. 2:**
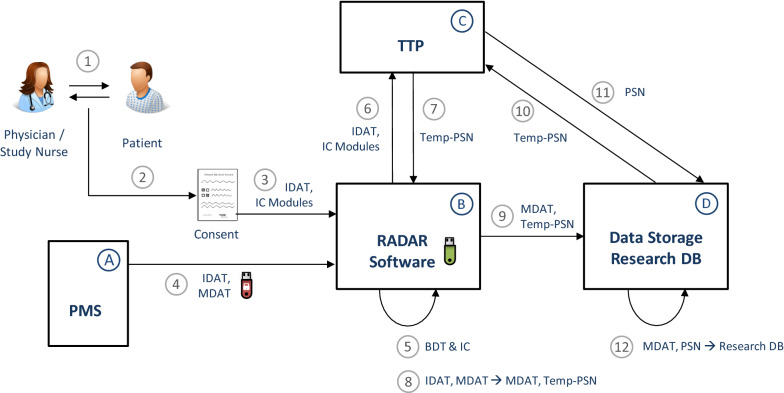
Workflow for data capture, transfer, and storage incl. building blocks

Figure 2 illustrates the workflow for data capture, transfer, and storage described in the following paragraph. Numbers (N) are used in the text to refer to the respective workflow parts in the figure. The mechanisms and workflows comply with the data protection concept drafted by the TMF (REQ #15).

Patient’s EHR data may be used for research purposes based on an informed consent given (GDPR Art. 6 lit. A; REQ #11). The data capture workflow starts with education of the patient regarding the consent (1), and the recording of the informed consent within the premises of the practice (REQ #1, #12). The consent is recorded on paper in RADAR (2). The consent is then documented digitally using manual input into the RADAR software (3) that comes with a graphical user interface (REQ #2, #5, #11). The consent text itself is module-based, i.e. study participation or re-contact are separate modules to be consented to. The consent documentation comprises IDAT and the patient’s consent for each module, thereby allowing specific automated consent queries for different purposes (i.e., consent modules). EHR data may be exported from the PMS and used for research purposes once the patient’s consent is given (REQ #11). EHR data export is done using the BDT export mechanism of the PMS (REQ #7, #3, #4), the export file is stored on a hardware-encrypted USB stick (4). The USB stick remains physically within the premises of the practice (REQ #15, #10). This USB stick is connected to a separate laptop that is not used for any practice-related works. On this laptop runs the RADAR software, which is delivered to the FP on a second unencrypted USB stick (REQ #2, #5, #10). The RADAR software validates the structure of the exported BDT data and selects the EHR data subset of the exported data for which a RADAR consent exists (5) (REQ #8, #14). The FP staff is shown an overview of the data ready to be sent. Once confirmed, data will be transferred through a secured (encrypted) network connection. For the selected EHR data subset, IDAT and consent information are sent to the TTP (6). As response, a temporary pseudonym for each patient is transferred to the RADAR software (7). The RADAR software replaces the former IDAT part using the temporary pseudonyms (8). The pseudonymised MDAT are then transferred to the data storage of project partner D (9), wherefrom the temporary pseudonyms are communicated to the TTP (10) to receive permanent, project-specific pseudonyms in return (11). As a result, MDAT are stored with the permanent pseudonyms in the research database (12) (REQ #15). After this, EHR data are processed, transferred, and stored as structured research data.

Search and retrieval of research data is another workflow supported in RADAR. Project partner D offers web-based access to the research database (REQ #17). Search criteria can be entered using a simple form. A full-text search is applied to the research database, the search result is presented as list of datasets that match the search criteria. The search results are downloadable as csv data file.

### Phase 3: implementing, testing and piloting

Based on the conceptual work from phase 2 a prototype of the RADAR software was implemented. The implementation itself was carried out in two steps: First, a BDT parser was implemented, which is able to import and process the BDT file format, and allows validation of BDT field lengths, data type, field syntax and the data set structure. Second, an interface module was coded, comprising the graphical user interface and connections to the RADAR data storage as well as the Trusted Third Party. Afterwards, the software passed the component tests successfully based on a set of prepared test data. The TTP core software modules for record linkage, consent management, and pseudonymisation were already implemented, coding efforts at the TTP comprised configuration and parameterisation of the workflows and interface. The interface used between data storage and TTP as well as between RADAR software and TTP is a technical REST interface, which is already successfully applied in other projects. The ‘temporary pseudonym’-based approach for data transfer from data source to research database using a TTP is re-used and almost identical to the implementation in other projects (REQ #15). [16, 17, 22, 23].

Data storage was implemented by defining a database layout for the BDT data elements and creating an instance of it. The data storage consists of a secured server, a MySQL database and an intermediate data storage area for MDAT files received from the RADAR software. Once a day, an automated procedure (i.e., a cron job) transfers the file-based MDAT to the MySQL database. The MDAT file is deleted afterwards.

Piloting the RADAR approach was also conducted in two steps: First, consented data sets of participating patients within participating FD practices were processed according to the designed workflows for data capture and storage. Every FP used a FP-specific password to initiate the data transfer between RADAR software and data storage. As a result, data sets of participating patients were successfully exported, separated into IDAT and MDAT, pseudonymised and saved in the RADAR data storage. This was done in one voluntary FD practice first, and then rolled out to eight FPs with 100 patients’ EHR data transferred into the research database. Second, the RADAR pilot was enhanced by designing a follow-up (FU) questionnaire to check if it was possible to link research results (i.e., items from filled-in questionnaires) with the existing medical data of the respective patients within the research database.

Some data processing steps described require passwords: one to unlock the hardware-encrypted USB stick, one to use the TTP interface for IDAT transfer, and another one to use the interface to the data storage to transfer the pseudonymised MDAT. Client certificates are used as second authentication factor for the TTP interface. Client certificates are provided by the TTP (REQ #15). The RADAR software used is coded in Java programming language in order to make a minimum of platform assumptions. It is provided on a second USB stick that is not encrypted. A separate notebook using a mobile network uplink to connect to the internet is also provided by the RADAR project team when visiting the FPs for data capturing. Therefore, no data processing or internet connectivity are required as a prerequisite for data capturing in a FP (REQ #2, #5, #9, #10). [24].

To involve patients in research, the Department of General Practice of the University Medical Center Göttingen developed the FU-questionnaires and defined selection criteria for those patients, who should receive the questionnaire. The selection was done using the search functionality of the research database. The result was a csv-formatted list of patients’ pseudonym (PSN) for the identified data items. PSN list and paper-based FU-questionnaire was sent as a request to the TTP to initiate the process of delivering the questionnaire to the respective patients because only the TTP has all information to re-identify these patients using their PSN. The TTP printed cover letters with name and address of respective patients, generated, stored the association with the research database PSN, and added an additional FU-PSN to each questionnaire. A stamped return envelope was added. The cover letter notified the recipients to not add their name, address, or signature to the returned questionnaires or the stamped return envelope. Then, letters were sent directly to respective patients. About two-thirds of the patients addressed sent back the filled-in questionnaire using the stamped return envelopes to the Department of General Practice of the University Medical Center in Göttingen, which digitalised the pseudonymised data and uploaded it to the data storage using the FU-PSN. To link a patient’s data set with respective FU data, the data storage exchanged the patient’s FU-PSN for the data storage PSN using the REST interface with the TTP. Record linkage was then completed successfully within the research database.

This enhanced pilot was successfully conducted using 63 questionnaire responses and demonstrated remarkably the automated data capture, storage of and, additionally, record linkage of research results (i.e. filled-in questionnaires) with the respective patient’s medical data in the RADAR research database.

## Discussion

BDT was defined for a different purpose than the one used for in RADAR. It has been used as best-possible approximation of the desired mechanism, as no modern PMS data exchange standard has been defined until now. No other data export options could be evaluated. Apparent shortcomings of the BDT export approach besides the outdated format were

- the barriers PMS vendors had installed to use the export mechanism (e.g., payment of one-time or per-export fees, purchase and installation of additional software, usage of daily-changed passwords that the vendor has to be asked for) and.
- non-compliance to the BDT specification, i.e. implementation-specific deviations that had to be considered when coding the BDT parser as part of the RADAR software. Examples of such deviations from the specifications are given in the following Table 2.

**Table 2:** Examples of deviations from the BDT specification found in exports

Field Code	Field name	Type	Length	Rules	Found content (Examples)
3110	Gender of the patient	Numeric field	1	Allowed content: 1 = male 2 = female	“M”, “W”, 0
6001	ICD code^a^	Alphanumeric field	5	Length $\le$ 5	Length > 5
8000	Sentence identification	Numeric field	4	Allowed content: 0010, 0020, 0021, 0022, 0023, 0101, 0102, 0103, 0104, 0190, 0191, 0199, 6100, 6200	“besa”
8410	Test Identification	Alphanumeric field	6	Length $\le$ 6	Length > 6

^a^International Classification of Diseases

As an effect of the above-mentioned shortcoming (a), participation in RADAR was limited to FD practices using one of two specific PMS that allow for free-of-charge data export. Based on the PMS inclusion criteria regarding free-of-charge data export other PMS could not be evaluated. As a result, RADAR software is tailored to those specific PMS and, therefore, may not be compatible with other PMS and their implementation-specifics. In addition, the focus on two specific PMS likely effected practice eligibility and participation in RADAR.

The issue regarding PMS-provided data export interface and mechanism can most likely be addressed and overcome by legislation only. The German legislator demands better interfaces for the subsequent use of treatment data. The modular design of the RADAR architecture is an important prerequisite for the later change of the interface used.

Architecture and workflows were influenced by the requirements listed in Table 1—including the organisational requirement no. 16 (REQ #16) which may have biased the solution. RADAR’s project setting and the developed architecture do not preclude any improved solution, but simply led to the described result. The results demonstrate feasibility to build a pseudonymised research data set while an anonymised data set may be achievable using different workflows.

The RADAR prototype proved the feasibility of (a) separating identifying and medical data early to comply with legal, organisational and technical requirements regarding data protection while (b) combining it with setting and processes in FDs’ practices. This data separation enables researchers to recontact RADAR participants for further data inquiry purposes without gaining knowledge of IDAT. The RADAR approach used two USB sticks—this approach was motivated by the need to limit possible requirements in respect to the FP’s infrastructure. Once more state-of-the-art options to extract and transfer data become commonly available in PMS, USB sticks as supplementary process elements could easily be exchanged for automated data transfer processes.

Similarly, the elimination of paper-based workflows, e.g. fully-digital consent recording is desirable. At the moment, paper-based consents need to be manually digitalised. This procedure consumes additional time of the FP’s staff and is prone to errors, e.g. typos. To reduce the burden on FP’s staff and to simplify research workflow integration in FD practices, a fully-digital consent recording is planned for the subsequent RADAR*plus* project.

## Conclusions

The RADAR project aims to design, implement, and pilot a generic research architecture and workflows to unlock primary care data for secondary use. The RADAR project builds on the conceptual results of earlier successful projects by developing an architecture together with a technical framework of tools, interfaces, and workflows for a complete infrastructure for practicable and secure processing of patient data from family doctors. The project partners used their many years of experience in their respective area of expertise to work out a solution. Core components of the architecture presented here include a common data privacy protection concept, the data protection-compliant management of patient’s identifying data and consent by the Trusted Third Party, the RADAR software used in the practices that intelligently examines and automatically processes BDT content and a research database that allows secure filing and analysis of the data obtained.

Architecture and workflows as technical components have been successfully implemented and demonstrated prototypically as working solution by the RADAR project. All technical components and workflows can be reused for further research projects. Practical benefits for the scientific community are apparent: Routine data from family practices is unlocked for research use, pooling of data with other databases is technically possible and depends primarily on the contents of the patient’s informed consent.

The RADAR project confirms the hypothesis that most technical components and processes as found in typical clinical research settings can be reused in a family doctor’s scenario. Additionally, a Trusted Third Party-approach can be used as core element to implement data privacy protection in such heterogeneous family doctor’s settings. However, family doctor’s settings differ significantly from hospital research settings regarding the prerequisites on which research architecture and workflows can be built upon. The majority of practices the RADAR project team had contacted do use a PMS, but a large technical diversity of PMS must be expected as counterpart for the research infrastructure. Data exports are mostly locked or otherwise unavailable. Once unlocked by license, data exports do not reliably conform to format standards.

The time practice staff needs for the research-related processes offers additional optimisation potentials. For example, fully-electronic consent recording is a much-desired goal and, therefore, part of the RADAR project’s extension phase.

The various architecture components of RADAR are reusable and do explicitly not rely on BDT as format or methodology; a replacement of the BDT parser is possible should the interface be changed. The KBV mandated such interface change in accordance with § 291d (1) SGB V. Consequently, all PMS vendors have to implement a new standardised interface. A respective update of the RADAR software is also part of the RADAR project’s extension phase.

## Data Availability

Study data are not to be disclosed according to the narrow RADAR consent.
